# Use of n-grams and K-means clustering to classify data from free text bone marrow reports

**DOI:** 10.1016/j.jpi.2023.100358

**Published:** 2024-01-04

**Authors:** Richard F. Xiang

**Affiliations:** Department of Pathology and Laboratory Medicine, Dalhousie University, Halifax, Nova Scotia, Canada

**Keywords:** Hematologic pathology, Bone marrow, K-means clustering, n-grams, Machine learning, Natural language processing

## Abstract

Natural language processing (NLP) has been used to extract information from and summarize medical reports. Currently, the most advanced NLP models require large training datasets of accurately labeled medical text. An approach to creating these large datasets is to use low resource intensive classical NLP algorithms. In this manuscript, we examined how an automated classical NLP algorithm was able to classify portions of bone marrow report text into their appropriate sections. A total of 1480 bone marrow reports were extracted from the laboratory information system of a tertiary healthcare network. The free text of these bone marrow reports were preprocessed by separating the reports into text blocks and then removing the section headers. A natural language processing algorithm involving n-grams and K-means clustering was used to classify the text blocks into their appropriate bone marrow sections. The impact of token replacement of numerical values, accession numbers, and clusters of differentiation, varying the number of centroids (1–19) and n-grams (1–5), and utilizing an ensemble algorithm were assessed. The optimal NLP model was found to employ an ensemble algorithm that incorporated token replacement, utilized 1-gram or bag of words, and 10 centroids for K-means clustering. This optimal model was able to classify text blocks with an accuracy of 89%, suggesting that classical NLP models can accurately classify portions of marrow report text.

## Highlights

• Natural language processing models utilizing n-grams and K-means clustering can accurately classify bone marrow text into their appropriate report sections.
• Model parameters such as data tokenization, n-gram number, and centroid number need to be optimized to achieve high model accuracy.
• The additional of a second K-means clustering model organized in an ensemble algorithm is able to further improve model performance.

## Introduction

Natural language processing (NLP) is a field of computer science aimed at utilizing computer algorithms to analyze language in an human-like way. NLP has been found to be useful in summarizing and interpreting diagnostic medical reports. In radiology, various computer algorithms such as Word2Vec, n-grams, continuous bag of words, and transformers have been studied in the tasks of disease classification, creation of cohorts for research studies, and assessing the compliance of scans with safety guidelines (reviewed in^1^^,^^2^).

NLP models involving convolutional neural networks have been shown to achieve high F1 scores in assigning International Classification of Disease for Oncology (ICD-O) codes to breast cancer reports.^3^ Another group has extracted the grade, stage, and quality of specimens from resections of bladder tumor by using hard coded NLP criteria.^4^ Automated summarization and machine translation of pathology reports have also been described in the literature (reviewed in^5^).

NLP has also be used to analyze bone marrow reports. The current state of the art NLP algorithms involves deep learning, and large language models (LLM), which were able to classify bone marrow report synopses with high accuracy.^6^ LLM provides a text prediction based on a block of input text. The unique feature of LLM is they are able to remember long blocks of text, and the ability to understand word meaning. This allows LLM to capture the relationship between words and the flexibility to interpret a variety of texts with minimal fine-tuning. However, large language model requires large text datasets and advanced computer hardware in order to train. Also, these LLM can suffer from bias if they are trained on data that is not accurately labeled.

One strategy to produce large quantities of accurately labeled text is to utilize another NLP model to preprocess and label the data. The labeling of this text can be achieved with classical NLP techniques, such as n-grams, bag of words, or manual rules based algorithms. Classical NLP algorithms provide the advantage of simpler model architecture, smaller training datasets, and lower hardware requirements. To our knowledge, there is only an abstract that employed a classical rules-based NLP algorithm to extract marrow and peripheral blood diagnoses and differential cell counts from bone marrow reports.^7^

Bone marrow reports are a suitable medical record for NLP studies because these reports are composed of different sections with guidelines on what information each section should contain.^8^ If the marrow section can be identified from a block of marrow text, then this would provide information about the contents of that text which would provide a foundation for further NLP learning and facilitate data organization. In this manuscript, we examined how NLP algorithms (such as n-grams and K-means clustering) were able to categorize the sections of hematological pathology reports. N-grams is a technique of splitting text into sequences of words. The number of words in each sequence is the value of n in n-grams. Splitting text blocks into word sequences allows an NLP algorithm to recognize word order and the frequency of how often that word sequence appears in text blocks. This information can then be used in a K-means clustering algorithm. In this manuscript, the K-means clustering algorithm is used as an automated method of grouping text blocks that are more alike based on the presence of different word sequences. Text blocks that contain the same sets of word sequences will be placed into the same group, and text blocks that contain different sets of word sequences will be placed into different groups.

We chose n-grams and K-means clustering due to their speed in training, smaller dataset requirement, and the easier ability to decipher the impact of different features compared to using other techniques, such as neural networks or LLM. We explored how altering the preprocessing of data, varying the number of n-grams and centroids, and utilizing an ensemble algorithm impacted the accuracy of our NLP model.

## Materials and methods

### Data acquisition

**Fig. 1:**
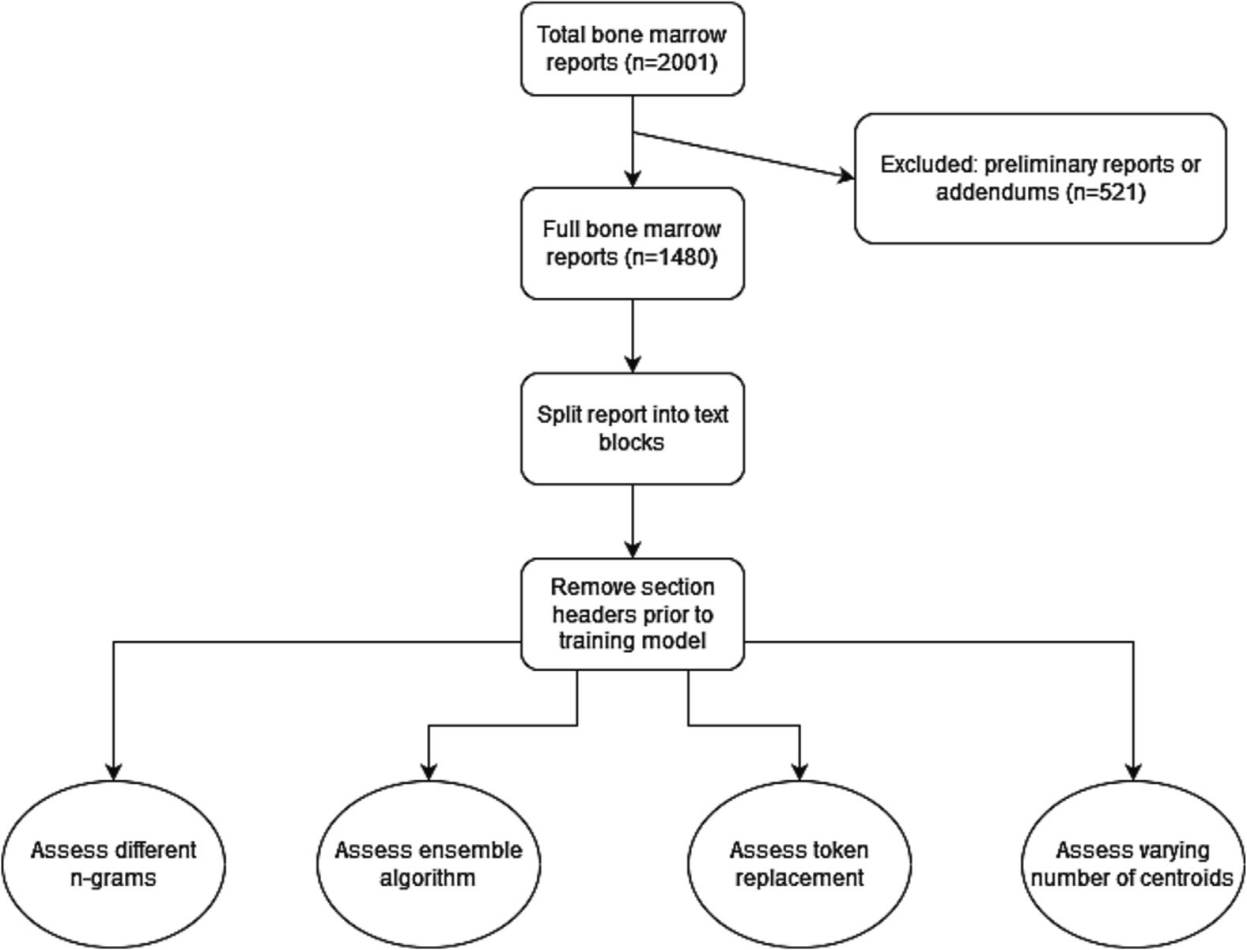
Diagram of data collection and analysis. Bone marrow reports were extracted as free text from the Nova Scotia Health laboratory information system. Only final bone marrow reports are included in this study and preliminary reports and bone marrow addendums were excluded. Each report was split into text blocks based on section headers and then these headers were removed prior to model training. The text blocks were then assigned report sections using a n-grams and K-means clustering model. The impacts of varying number of n-grams, varying the number of centroids, utilizing an ensemble algorithm, utilizing token replacement on the performance of this model were assessed.

The lab information system of Nova Scotia Health Central Zone was queried for 2001 in-house bone marrow reports from June 2021 to May 2023. Preliminary and addendum reports were excluded. Patient identifiers were removed and the anonymized reports were copied and pasted as free text and stored in a spreadsheet (Fig. 1). Ethics approval was obtained from the Nova Scotia Health Research Ethics Board (file #1028847).

### Natural language processing

The computer algorithms to preprocess the bone marrow reports, separate the text into n-grams, perform K-means clustering, and implement an ensemble algorithm were written in the Python programming language, version 3.9.13.^9^ Numpy version 1.21.5,^10^ sklearn version 1.0.2,^11^ nltk version 3.7,^12^ and pandas version 1.4.4^13^ python libraries were used.

### Data preprocessing

The free text of bone marrow reports were split into text blocks based on the section headers (specimen received, clinical information, peripheral blood smear, bone marrow aspirate, bone marrow aspirate differential, iron content, bone marrow biopsy, comment, diagnosis, and disclaimer). The bone marrow reports were then split into training and testing subsets (80:20 ratio, respectively). This split was based on chronology with the older bone marrow report placed in the training dataset while the newer reports were placed in the testing dataset. Prior to model training or testing, the section headers were removed from the text blocks.

### Replacement of related n-grams with token and assessment of n-gram number

The presence of accession numbers, cluster of differentiation (CD), or numerical values could provide information about the marrow section that a text block belonged to. However, different accession numbers were represented by different texts, and the algorithm was unable to recognize that these different texts are all accession numbers. In order to allow the algorithm to recognize this information, accession numbers needed to be replaced with a term that was exactly the same for all accession numbers. This is called a token. This same process was performed for CD values and numerical values. Using an automated process, the bone marrow report text that had accession numbers, cluster of differentiation, and numeric values were replaced with $\langle \text{ACCESSION NUMBER}\rangle$, $\langle \mathrm{CD}\;\text{NUMBER}\rangle$, or $\langle \text{NUMBER}\rangle$ tokens, respectively. Both the original bone marrow reports and the reports with the replacement tokens were processed into 1 to 5 grams and used to create training and testing datasets.

### K-means clustering

Each text block was converted into a feature vector where each element was a binary value that tracked whether a specific n-gram was present at least once in the text block. These feature vectors were then used to train a K-means clustering model. K means clustering was repeated 10 times with randomized centroid initiation and the run with the lowest sum of the squared errors (SSE) was chosen as the trained model. The trained models were used to predict the bone marrow report section that the text blocks in the testing dataset belonged to. In order to use K-means clustering to assign bone marrow sections, we determined which section was most common in a cluster and then assign all text blocks in that cluster to that most common section.

### Core model

The core model to compare the impact of different n-grams and K-means clustering parameters was established by replacing the accession numbers, cluster of differentiation (CD) numbers, and numerical values with tokens. The core model utilized the 200 most frequently occurring 2-grams and performed K-means clustering with 10 centroids. The performance of the core model was compared to models where different n-grams were used (from 1-gram/bag of word to 5-gram), where a different number of clusters were used in K-means clustering (1–19), where the accession number, CD numbers, and numerical values were not converted to unique tokens, and where a second K-means clustering model was utilized in an ensemble algorithm.

### Effects of varying the number of clusters

Using the bone marrow reports with token replacement, K-means clustering was trained with different numbers of centroids, varying from 1 to 19. The trained models were then used to predict the testing dataset, and the SSE was calculated. The SSE versus number of centroids was graphed for 1-gram and 2-gram and the elbow method (as described in^14^) was employed to determine the inflection point of the graph. K-means clustering models using the number of centroids specified by the inflection point were compared to the core model using 10 centroids.

### Assessing model performance

**Fig. 2:**
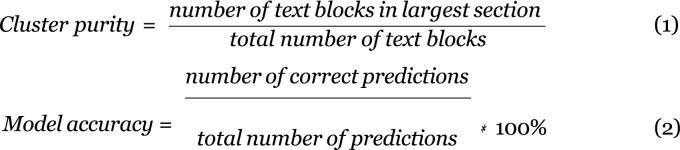
Equations for cluster purity and model accuracy.

The performance of a K-means clustering model was assessed by 2 metrics (Fig. 2). The first metric will be referred to as cluster purity and was calculated separately for each cluster. Cluster purity was calculated by first determining which marrow report section had the largest number of text blocks, then recording the number of text blocks in that section and dividing by the total number of text blocks in the cluster. The second metric was the accuracy of a model. The accuracy was calculated by dividing the number of correct predictions by the total number of predictions from a model. A correct prediction is a text block where the marrow section predicted by the clustering model matches the marrow section where the text block was extracted from (i.e., the true marrow section).

### Ensemble model

An ensemble K-means clustering algorithm was tested by first training a K-means clustering model with 2-gram. Then, the 2-gram model was used to predict the text blocks of the testing dataset. This separated the text blocks into 10 different clusters. Next, the text blocks in the cluster with the lowest cluster purity were selected as the input dataset for additional sorting by a second K-means clustering model. This second model clustered the text blocks based on number of numeric values in the text block, median paragraph length in the text block by word count, and the relative location of the text block in the marrow report. This second K-means model utilized 5 centroids.

### Statistical analysis

Chi square testing was performed on our core model, with the expected outcome that there would be an evenly distribution of text blocks among the report sections within each cluster.

## Results

### Effects of replacement with tokens and altering n-gram number

**Fig. 3:**
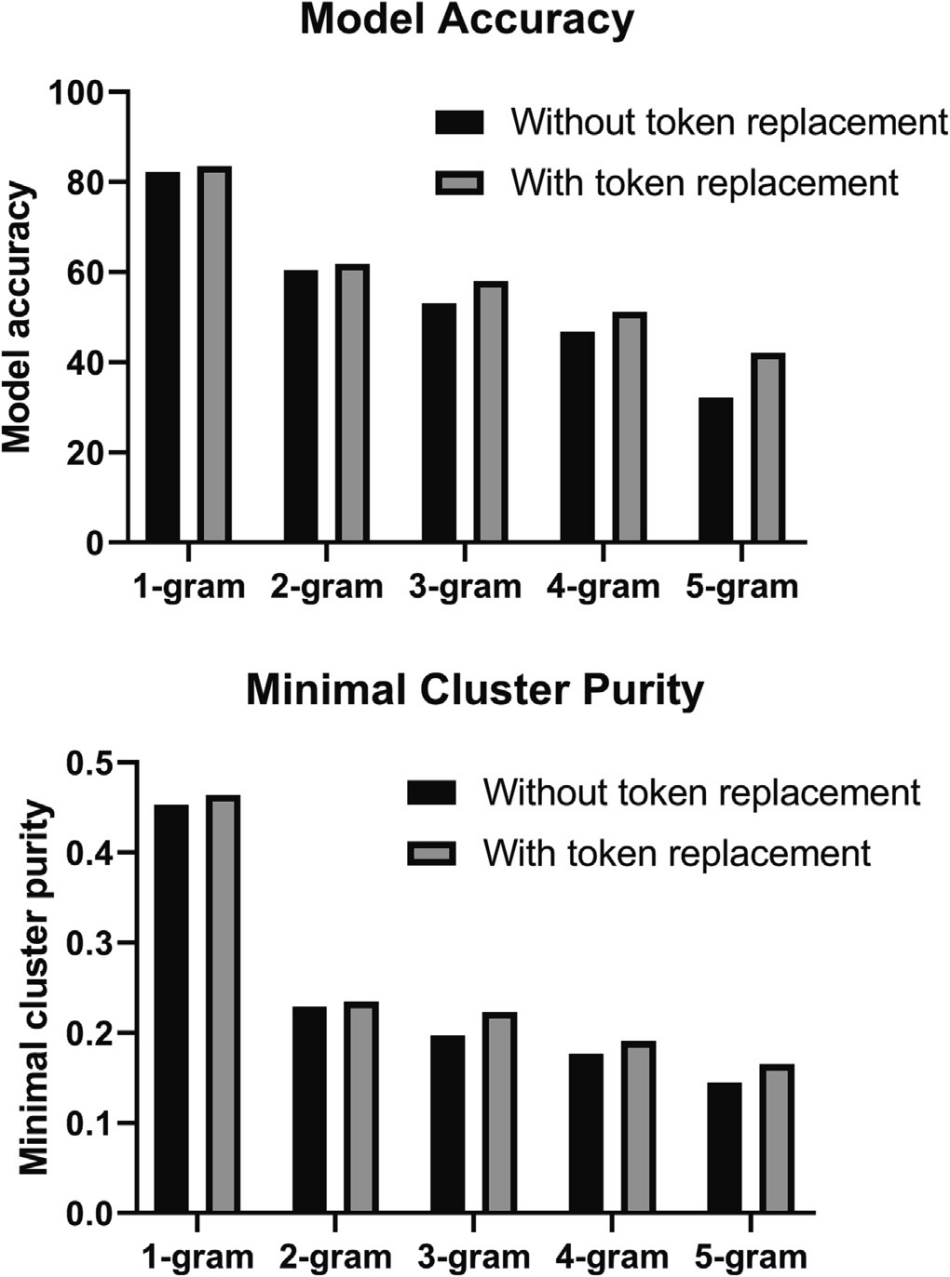
Replacement of accession numbers, clusters of differentiation, and numeric values with tokens improved model performance. This figure shows bar graphs comparing K-means clustering using 1 to 5-gram and either without token replacement (black bars) or with token replacement (gray bars). Top: Compares model accuracy. Bottom: Compares the minimal cluster purity.

The effect of replacing similar words/numbers with tokens improved the accuracy of the K-means clustering model for all n-grams tested. The percent improvement was 1.4% for 1-gram and this increased to 9.9% for 5-gram (Fig. 3). Token replacement also improved the minimal cluster purity, which was the lowest purity of all the clusters in a specific model. The improvement in the minimal cluster purity varied between 0.006 and 0.02. The minimal cluster purity shows a large decrease between 1-gram/bag of words and 2-gram, while the decreases from 2-gram to 5-gram were much smaller. Fig. 3 also shows that lower n-gram numbers had better accuracy and minimal cluster purity. The highest accuracy was 83.6% with a minimal cluster purity of 46.4%, achieved by a model utilizing 1-gram and token replacement.

### Effects of varying the number of clusters

**Fig. 4:**
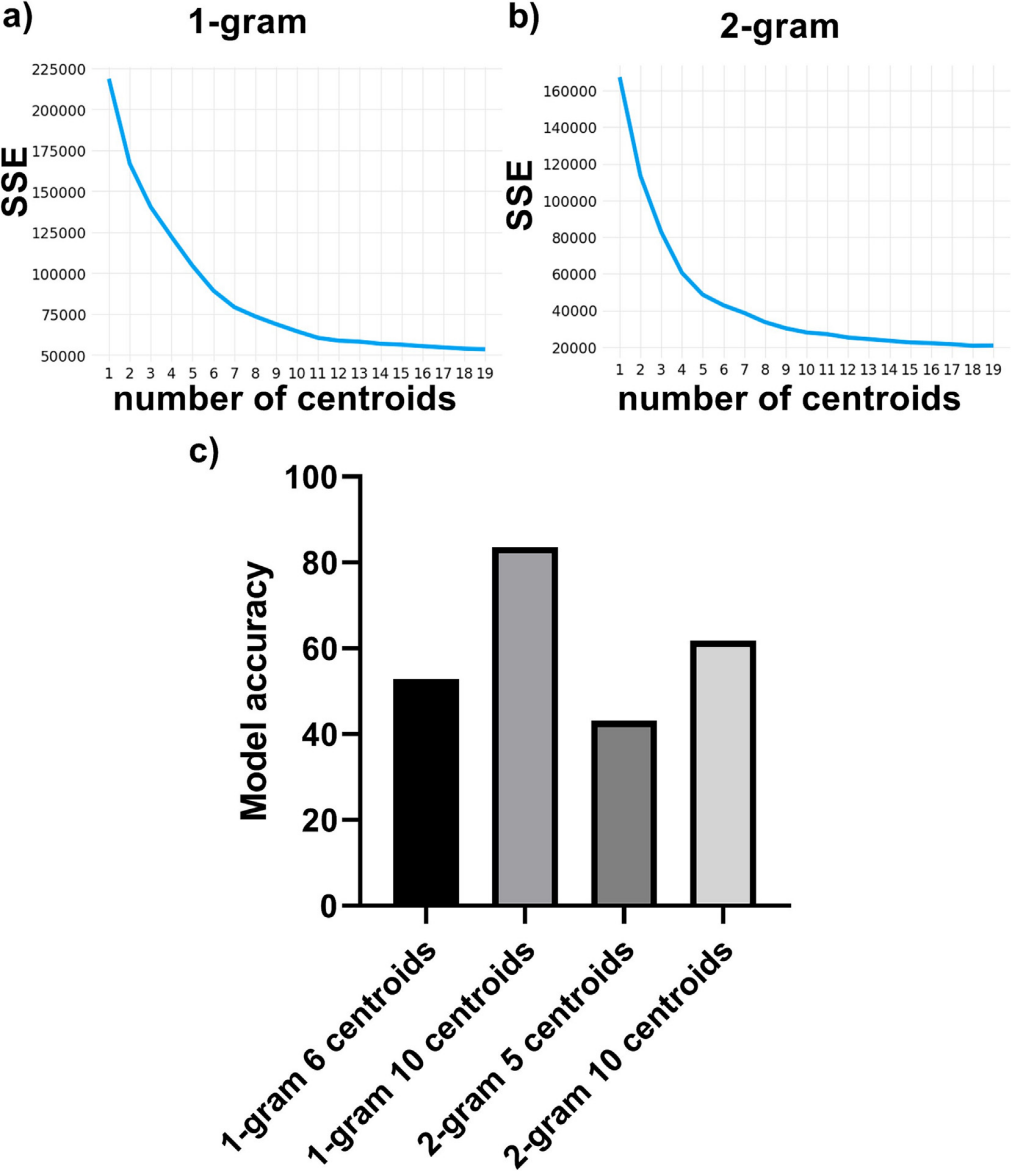
A K-means clustering model using 10 centroids was more accurate than using the number of centroids based on elbow method. The SSE was plotted against the number of centroids used to train the 1-gram (a) or 2-gram (b) models. The K-means clustering models were then used to predict the correct report section for the text blocks in the training set. (c) Bar graph showing model accuracy for the 1-gram and 2-gram models with different number of clusters.

Plotting the SSE versus number of centroids revealed an inflection point for the 1-gram model at 6 centroids and the 2-gram model at 5 centroids (Fig. 4a and b). The model accuracies were better when 10 centroids were used, compared to when using the elbow method to determine the number of centroids. For the 1-gram model, the model accuracy was 52.8% and 83.6% for 6 and 10 centroids, respectively. For the 2-gram model, the model accuracy was 43.1% and 61.8% for 5 and 10 centroids, respectively. Performing chi square testing of the core model (2-gram with 10 centroids) showed statistical significance compared to assigning the text blocks in an equal distribution to each report section (*P* .00001).

### Effects of ensemble algorithm

**Fig. 5:**
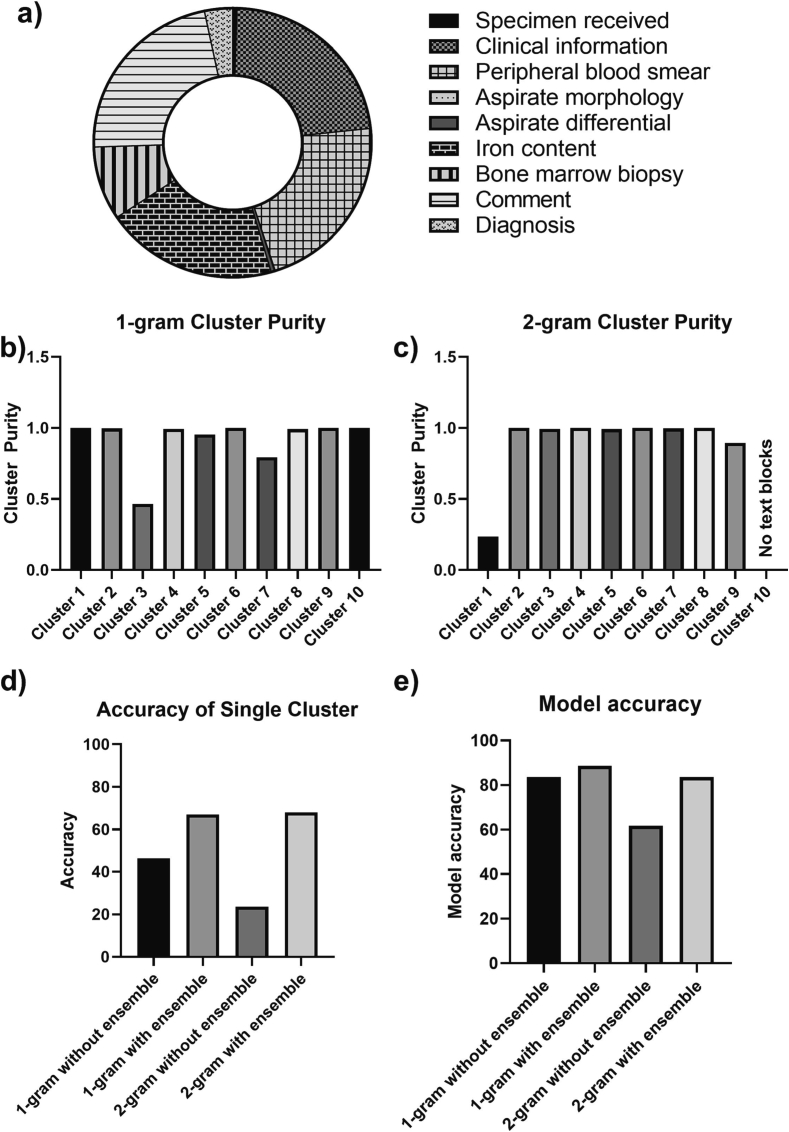
Ensemble K-means clustering model improved model accuracy. The text blocks from the cluster with the lowest purity were recorded. (a) Pie chart showing the report sections that composed the cluster with the lowest purity of the 2-gram model. (b) and (c) Bar graphs showing that 1 cluster has much lower purity in the 1-gram (b) and 2-gram (c) models. There were no text blocks assigned to cluster 10 in the 2-gram model, so it was excluded. (d) Bar graph assessing the accuracy of the lowest purity cluster with and without an ensemble model. (e) Bar graph comparing how the ensemble model improved the overall accuracy of the 1-gram or 2-gram models.

Fig. 5 shows that text blocks in the cluster with lowest purity in the 2-gram model was composed mainly of text from the marrow report sections: clinical information (21.75%), peripheral blood smear (21.75%), iron content (20.11%), and comment (22.24%). Both 1-gram and 2-gram models had a single cluster which demonstrates a much lower cluster purity when compared to the other clusters (Fig. 5b and c). The purity of the lowest and second lowest clusters for 1-gram are 0.46, and 0.79, while for 2-gram, they are 0.23 and 0.89, respectively. The addition of a second model was able to improve the accuracy of the lowest purity cluster of the 1-gram model to 67% and the 2-model to 68%. By improving the accuracy of the lowest purity cluster, the overall accuracy of the 1-gram and 2-gram models improved to 88.6% and 83.5%, respectively.

### n-gram frequency distribution

Table 1 shows how frequently the most common 100 n-grams appear in the marrow text. The n-grams for the 1-gram model occur more frequently than the n-grams for the 2-gram model.

**Table 1:** Most frequently appearing n-grams in marrow reports.

1-gram	Frequency
$\langle \mathrm{NUM}\rangle$	21 139
and	8161
of	7852
is	6524
are	5592
$\langle \mathrm{LETTER}\_\mathrm{NUM}\rangle$	5159
the	4831
with	4795
in	4586
BONE	3734
MARROW	3726
a	3280
marrow	2863
for	2851
to	2682
cells	2633
$\langle \mathrm{CD}\#\#\rangle$	2608
A	2290
No	2282
The	2211
bone	2168
-	2007
cm	1948
seen.	1876
Bone	1842
ASPIRATE	1810
B	1802
This	1681
Description	1601
not	1577
Marrow,	1497
was	1492
by	1482
Plasma	1451
Aspirate	1439
an	1321
or	1321
specimen	1214
core	1197
biopsy	1184
at	1181
seen	1172
plasma	1170
Biopsy	1139
no	1131
There	1118
on	1064
this	1031
increased	1016
AND	985
Specimen:	952
as	951
significant	940
BIOPSY:	917
M:E	914
iron	911
cell	905
Normal	899
stain	897
present.	883
x	882
Lymphocytes	870
smear	862
there	856
Peripheral	854
be	845
Smear	844
None	843
Specimen	841
Clinical	841
Lymphocytes:	841
Pathologist	838
Gross	837
Cellularity:	836
IRON	827
Blood	821
Quality	821
blasts	814
Received	803
Microscopic	802
Diagnosis	802
Information	801
(Electronic	801
Signature)	801
Verified:	801
$\langle \mathrm{END}\_\mathrm{OF}\_\mathrm{REPORT}\_\mathrm{TOKEN}\rangle$	800
Comment	797
In	797
DIFFERENTIAL	796
slides	791
Neutrophils	790
CONTENT	790
stain.	788
Superior	780
Iliac	780
Ratio:	780
Approx.	777
stained	776


2-gram first term	Second term	Frequency
$\langle \mathrm{NUM}\rangle$	%	6638
BONE	MARROW	3502
$\langle \mathrm{NUM}\rangle$	cm	1942
bone	marrow	1846
MARROW	ASPIRATE	1645
Bone	Marrow,	1497
$\langle \mathrm{NUM}\rangle$	$\langle \mathrm{NUM}\rangle$	1405
with	a	1248
at	$\langle \mathrm{NUM}\rangle$	1056
$\langle \mathrm{LETTER}\_\mathrm{NUM}\rangle$	$\langle \mathrm{LETTER}\_\mathrm{NUM}\rangle$	1004
are	not	876
There	is	823
and	an	807
Clinical	Information	801
Peripheral	Blood	801
Microscopic	Description	801
Pathologist	(Electronic	801
(Electronic	Signature)	801
Signature)	Verified:	801
Verified:	$\langle \mathrm{NUM}\rangle$	801
Specimen	Received	800
Gross	Description	800
Blood	Smear	799
$\langle \mathrm{END}\_\mathrm{OF}\_\mathrm{REPORT}\_\mathrm{TOKEN}\rangle$	Specimen	799
Description	BONE	797
A	Bone	796
IRON	CONTENT	790
Quality	of	789
of	Specimen:	788
ASPIRATE	DIFFERENTIAL	787
Received	A	781
Superior	Iliac	780
M:E	Ratio:	778
Approx.	M:E	777
Lymphocytes	$\langle \mathrm{NUM}\rangle$	776
$\langle \mathrm{NUM}\rangle$	x	776
slides	are	775
Total	cells	775
cells	counted:	775
counted:	$\langle \mathrm{NUM}\rangle$	774
there	is	774
A	-	772
stain	and	772
an	iron	772
iron	stain.	772
-	Aspirate	771
Aspirate	smear	771
smear	slides	771
are	prepared	771
prepared	and	771
and	stained	771
stained	with	771
conventional	bone	771
marrow	stain	771
$\langle \mathrm{NUM}\rangle$	IRON	771
a	conventional	770
Plasma	Cells:	769
%	Lymphocytes	767
CONTENT	Hemosiderin:	766
Neutrophils	$\langle \mathrm{NUM}\rangle$	763
Seg.	Neutrophils	762
%	Total	762
on	the	761
ASPIRATE	Quality	756
Erythroblasts	$\langle \mathrm{NUM}\rangle$	750
Posterior	Superior	745
%	Erythroblasts	745
MARROW	BIOPSY:	741
of	the	729
%	Seg.	728
Monocytes	$\langle \mathrm{NUM}\rangle$	722
%	Monocytes	716
Myelocytes	$\langle \mathrm{NUM}\rangle$	713
FIXATIVE:	Formalin	703
Formalin	NO.	703
NO.	PIECES:	703
Description	A	702
Ratio:	$\langle \mathrm{NUM}\rangle$	700
Individual	Performing	685
Performing	Grossing	685
x	$\langle \mathrm{LETTER}\_\mathrm{NUM}\rangle$	684
of	this	676
Metamyelocytes	$\langle \mathrm{NUM}\rangle$	673
$\langle \mathrm{NUM}\rangle$	Erythropoiesis:	672
Other	Elements:	668
%	Metamyelocytes	667
Diagnosis	BONE	660
B	Bone	658
plasma	cells	658
This	case	657
Marrow,	Biopsy	655
case	was	653
Marrow,	Aspirate	650
was	grossed	649
grossed	by	649
for	decalcification	643
Grossing	This	643
CASSETTES:	In	641
Iliac	Spine)	631

## Discussion

To assess model performance, we used cluster purity and model accuracy. A high cluster purity would have provided a high confidence that a text block in a specific cluster was correctly assigned. A high accuracy would have suggested that a model would have a high likelihood of assigning a text block to the correct report section. It was possible to have had a model with high accuracy but have one or more clusters in that model with low cluster purity. This would have occurred if a cluster had low purity but was also rarely utilized by the model and rarely were text blocks assigned based on that cluster. This can be seen in Fig. 5b, where the 1-gram model without an ensemble algorithm had a high accuracy of 84% but also contained a low cluster with a purity of only 46%.

Preprocessing the bone marrow text by replacing accession numbers, numerical values, and clusters of differentiation with respective tokens improved the model accuracy and minimal cluster purity (Fig. 2). This was an expected result since bone marrow reports are standardized to contain specific sections including: specimen received, aspirate differential, and immunohistochemistry. The specimen received section often contained the accession number of the bone marrow specimen. Similarly, the aspirate differential contained a large quantity of numerical values, and the immunohistochemistry section mentioned clusters of differentiation. The replacement with respective tokens allowed the K-means model to recognize these sections.

In other natural language processing algorithms, the preprocessing step would also convert the case of all letters to either upper or lower case.^15^ We considered implementing this additional step. However, when we examined our marrow reports, we found that the diagnostic line often used fully capitalized letters and the diagnosis in the comment section often had the first letter for each word capitalized. Therefore, we wanted to preserve the capitalization of the letters since they could have provided information about which section the text block was in.

Since the training and testing datasets are split based on chronology, we were able to assess if training a K-means clustering model on a dataset comprised of older bone marrow reports would be able to classify text of newer bone marrow reports. In Fig. 3, we found that this was possible since our trained model had an high accuracy of 83.6%. This finding suggested that our NLP model would not have to be re-trained whenever a set of bone marrow text needed to be classified. The ability to pre-train a model will save time and reduces the complexity of using the model, since datasets would not have to merge older and newer reports together prior to K-means clustering and models would not have to be re-trained.

Analysis of the impact of different number of n-grams (Fig. 2) revealed that lower n-gram numbers produced more accurate models, with 1-gram or bag of words model producing the best model in terms of accuracy and minimal cluster purity. Table 1 shows that the frequency of the 100 most common 1-grams occurred more often than the 100 most common 2-grams. This finding provided an explanation for the improved performance seen with the 1-gram model. The higher frequency allowed more text blocks to contain the n-grams, which then allowed more positive features for the K-means clustering model to separate the clusters by. This suggested that for bone marrow reports, the presence of n-grams was a limiting factor to the performance of K-means clustering models.

A common method of determining the optimal number of centroids to utilize in K-means clustering is based on the SSE.^14^ This method involves plotting the SSE versus the number of centroids and choosing the number of centroids where the “elbow-shape” is located.^14^ In Fig. 4, we examined how SSE decreased with increasing number of centroids. An elbow shape was seen with both the 1-gram and 2-gram models, with the elbow shape appearing at 6 centroids for the 1-gram model and 5 centroids for the 2-gram model. At our institution, we commonly used 10 bone marrow sections, and therefore we tested if using 10 centroids would have provided more accurate models than if we based the centroids off the elbow method. Fig. 4 showed that utilizing 10 centroids led to a higher model accuracy, which suggested that basing the centroid number on the number of bone marrow sections was a valid approach.

When the purity of the clusters for 1-gram and 2-gram models were calculated, there was one cluster that was noticeably lower than the other clusters (Fig. 4b and c). This raised the question if the accuracy of these low purity clusters could be improved if analyzed by another K-means clustering model with a different set of features. In order to determine which features to assess in the second model, marrow report sections that were most commonly in the low purity clusters were assessed and found to be mainly the comments, clinical information, iron content, and peripheral blood smear sections (Fig. 5a). Therefore, we utilized the number of numerical values, which would help separate the peripheral blood section which contained the complete blood count (CBC) indices. We also included the median paragraph length, since the iron content section had fewer words than other marrow sections. We also included the relative location of the text block, since the clinical information section was usually at the start of the report and the comments section was at the end. These 3 factors were used as the features of a second model to analyze the text blocks of the lowest purity clusters from the 1-gram and 2-gram models. This second model improved the accuracy for the lowest purity clusters, which in turn improved the model accuracy of the ensemble model compared to the n-gram model (Fig. 5d and e). This suggested that additional features can be incorporated into an n-gram model to improve performance and that these features can be include through an ensemble approach.

In this manuscript, we demonstrated that our model is able to accurately assign bone marrow report sections to marrow report text. This model could be incorporated directly in to a laboratory information system to ensure that reports have all required sections before they are finalized. The ability to assign sections to marrow text can also assist in organizing the electronic storage of marrow reports by separating the full text of bone marrow reports into subsections. This model could also label large datasets of marrow text, which could be utilized to train other NLP models, such as LLM.

Our study has some limitations, mainly due to the dataset used, the n-gram algorithm, and the ensemble model. The first limitation is that this study only utilized bone marrow report data from a single healthcare network, and the hematological pathologists within that network may have a similar style of reporting. It is expected that this model would need to be retrained for bone marrow reports extracted from a different healthcare network’s laboratory information system. Additionally, if there is excessive variability in the formatting of reports, then the accuracy of the trained model will be decreased. Also, we only tested English marrow reports. It is possible that marrow reports in a different language with a different grammatical structure would not carry the same information when separated by n-grams. Another limitation with using n-grams is the requirement of containing enough text, so that the common n-grams are present. This raises the possibility of a minimum word count for the text blocks and suggests our model would have poor performance if used to classify short text blocks. Our ensemble approach also has specific limitations. The degree of improvement in model accuracy from the ensemble approach is limited based on the text blocks in the cluster with the lowest purity from the first K-means clustering model. If there are traits that allow subdivision of these text blocks, then a large improvement in accuracy can be attained from the addition of a second K-means clustering model. However, it is possible that there are no traits to subdivide the text blocks, which will limit the improvement from an ensemble approach. In our ensemble approach, the order of marrow report sections in the training dataset is important. If another institute has a different way of ordering their marrow report sections, then the ensemble model will have to be trained on reports from that institution to achieve the optimal accuracy.

## Conclusion

We studied the use of n-grams and K-means clustering to assign blocks of bone marrow text to their appropriate report section. We found several factors that improves the model’s accuracy, including replacement of numerical values, accession numbers, and CD values with tokens, using low number n-grams, using the same number of centroids as there are bone marrow sections, and utilizing an ensemble K-means clustering model. By optimizing all of these factors, we were able to achieve a model that was highly accurate in classifying text blocks extracted from the free text of bone marrow reports. An accurate algorithm to assign the marrow sections could allow for automated tagging of bone marrows reports with descriptive notes, verification that marrow reports are complete, and text extraction for future machine learning projects. This manuscript can help to be a foundation for future NLP research in hematologic pathology.

## Declaration of competing interest

The authors declare that they have no known competing financial interests or personal relationships that could have appeared to influence the work reported in this paper.
